# The skin temperature under multilayered bandages during exercise

**DOI:** 10.1080/07853890.2021.1896730

**Published:** 2021-09-28

**Authors:** Paula Cardoso, Ana Paixão, Margarida Claro, André Pinto, Ângela Pereira

**Affiliations:** a Instituto Português de Oncologia de Lisboa, Lisboa, Portugal; b Department of Physiotherapy, Escola Superior de Saúde Egas Moniz (ESSEM), Egas Moniz Cooperativa de Ensino Superior, Caparica, Portugal; c Centro de Investigação Interdisciplinar Egas Moniz (CiiEM), Egas Moniz Cooperativa de Ensino Superior, Caparica, Portugal; d Hospital Garcia de Orta, Almada, Portugal; e Hospital de Cascais Dr. José de Almeida, Cascais, Portugal; f Oxleas NHS Foundation Trust, London, UK

## Abstract

**Background:**

MultiLayer Bandages (MLB) assume a prominent role in the physical treatment of lymphedema, being an integral part of decongestant lymphatic therapy. This, according to the International Society of Lymphology, consists of a reduction phase and a maintenance phase and includes procedures such as: hygiene care, manual lymphatic drainage, multi-layered bandages, exercise, and elastic compression [1]. Although MLBs are widely used in the treatment of lymphedema, and the physical principles underlying their therapeutic efficacy are well known [2], the mechanism of action regarding the thermal effect has not been clearly defined. When applied, they cause an rise in skin temperature [3], and during exercise this temperature further increases due to the intensification in metabolic activity and heat production [4,5], but there is no actual data regarding the temperature values and its variation (3). The aim of this study was to evaluate the cutaneous temperature of the upper limb when submitted to the application of MLB during physical exercise of moderate intensity [4], and clarify if the temperature is an obstacle to its use, when compared to the upper limb skin reference value.

**Methods:**

A quasi-experimental study was designed with a sample of 30 individuals (without known pathology). All participants signed an informed consent and the study followed all the principles of the Declaration of Helsinki. The individuals were submitted to the application of a temperature-bioPLUX research sensor in the external region of both forearms. The experimental group (EG) was considered to be the dominant upper limb submitted to MLB application and the control group the contralateral upper limb. The temperature was recorded in 4 evaluation moments: at rest, before and after MLB application, during exercise on an elliptical trainer (until the individual reached 69% of the Maximum Heart Rate) and after cessation of exercise (when the rest Heart Rate was reached). Descriptive statistics were used to characterise the sample, using inferential statistics to analyse the research questions. The results were considered significant at the significance level of 5% and the statistical program SPSS v23.0 was used.

**Results:**

At the first evaluation moment of at rest and without band, no statistically significant differences between the groups were detected. The experimental group had 32.8 ± 0.85˚C and the control group 32.7 ± 0.81˚C (*p* = .088). With MLB application at rest (second evaluation moment) and during exercise (third evaluation moment), there was a statistically significant difference between the groups (*p* < .001). At the last evaluation moment (post-exercise) the temperature of the experimental group continued to increase to 35.0 ± 0.67˚C, whereas the control group decreased to 32.8 ± 0.90˚C, and a statistically significant difference was observed (*p* < .001). Despite the increase observed in the EG, the value did not exceed the skin reference value of the upper limb [4,6].

**Discussion and Conclusions:**

The results obtained allow us to conclude that with the application of MLB the skin temperature raises, which agrees with the results of Belgrado [3], who states that the increase in the skin temperature under MLB occurs minutes after its application. With exercise, it is observed that the skin temperature continues to rise justified by the intensification in metabolic activity, being necessary to activate the hypothalamic thermoregulation system [7]. When physical exercise ceases, the cutaneous temperature of the upper limb with MLB continues to rise due to the inability to release heat. Lim [6] and Campbel [8] state that if heat dissipation is not carried out effectively, heat builds up and increases body temperature. Despite this increase in temperature, the maximum values collected during the study did not exceed the skin reference temperature of the upper limb for individuals without pathology (35.5˚C) defined in the literature [6,9]. Therefore, the temperature was not considered as an obstacle in the use of MLB for the population in study.
